# Canadian dental hygienists’ experiences and perceptions of regulatory guidelines during the COVID-19 pandemic: a qualitative descriptive analysis

**DOI:** 10.1186/s12913-022-08925-z

**Published:** 2022-12-22

**Authors:** Lindsay K. Macdonald, Michael Glogauer, Paul Allison, Carlos Quiñonez, Sreenath Madathil, Leigha D. Rock

**Affiliations:** 1 Faculty of Dentistry, Dalhousie University, 5981 University Avenue, Room, Halifax, 5224 Canada; 2 Faculty of Dentistry, University of Toronto, Toronto, Canada; 3 Faculty of Dental Medicine & Oral Health Sciences, McGill University, Montreal, Canada; 4 Schulich School of Medicine and Dentistry, Western University, London, Canada; 5 Department of Pathology, Faculty of Medicine, Dalhousie University, Halifax, Canada

**Keywords:** Dental hygienists, Canada, COVID-19, Regulatory body, Guidelines, Protocol, Communication, Messaging

## Abstract

**Background:**

In Spring of 2020, due to the COVID-19 pandemic, Canadian provincial dental hygiene regulatory bodies implemented new practice guidelines. Reports of stress, anxiety and conflict experienced by dental hygienists have been linked to miscommunication between oral health regulators at this time. Limited data exists on the perceptions and experiences of dental hygienists navigating new guidelines for dental hygiene care during the pandemic. Therefore, the objective of our study was to explore via descriptive thematic analysis how dental hygienists experienced and perceived: i) dental hygiene practice during the COVID-19 pandemic, and ii) their regulatory body’s COVID-19 guidelines.

**Methods:**

Participants were identified through provincial dental hygiene licensing bodies. Online bi-monthly questionnaires were administered to participants (*n* = 876) from December 2021 to January 2022. Two open-ended questions were asked in the questionnaire. A qualitative descriptive thematic analysis was applied to these two questions.

**Results:**

Major themes at baseline relayed challenges related to workplace compliance, patient treatment and communication of practice protocols. Across responses, hygienists confirmed conflicting messaging from regulators and guideline interpretations as stressors impacting their professional practice and satisfaction within the profession. Participant responses at endpoint cited increased satisfaction with regulatory guidelines as the pandemic evolved, yet inconsistencies in regulators’ messaging was noted as a prevailing issue.

**Conclusion:**

Inconsistent guideline messaging reflects an increased need for collaboration amongst oral health care regulators to streamline protocols for practice and reduce interprofessional conflict in pandemic circumstances. A national unified approach is warranted in establishing guidelines for dental hygiene practice in Canada.

**Supplementary Information:**

The online version contains supplementary material available at 10.1186/s12913-022-08925-z.

## Background

In Spring of 2020, oral health services in Canada experienced significant interruption as a result of the unprecedented epidemiology of the SARS-CoV-2 (COVID-19) virus [1, 2]. Following nation-wide furloughs of non-essential services including non-emergency oral health care, as increased scientific evidence on COVID-19 transmission was confirmed, provinces reopened to the provision of dental hygiene services in the Summer of 2020 [3]. To satisfy reopening standards set by federal legislation and provincial public health officials, provincial regulatory colleges for dental hygienists were required to develop and implement new practice guidelines for patient care [3]. The basis of rationale for new guidelines was to calibrate clinical practice with the most current available evidence on COVID-19. This included compliance with new best practice standards across the spectrum of personal protective equipment (PPE), use of AGPs (aerosol-generating procedures), social distancing measures and screening processes for potential SARS-CoV-2 infection in patients [3–6].

Recently, the evidence available has identified detrimental impacts of the COVID-19 pandemic on the occupational health and safety of practicing health care professionals [7, 8]. For oral health care providers (OHCPs), identified as a high-risk group for SARS-CoV-2 infection due to their intimate involvement with the oral cavity, studies have concentrated on examining incidence rates of infection [5, 9, 10]. This includes researchers in this study who previously examined incidence rates of infection amongst Canadian dentists [10]. Complementary to our present study, previous research has identified a subsequent gap in knowledge pertaining to incidence of infection amongst Canadian dental hygienists [10]. The aims of our present study however are unique in that we sought to examine incidence rate as well as perceived anxiety experienced by dental hygienists during the evolution of patient care practices during the pandemic. Currently, there is little substantiated evidence available to inform the perceptions, anxiety, and experiences of dental hygienists in navigating communication of new regulatory guidelines for dental hygiene services during the pandemic [6]. However, anecdotal evidence and media reports from this period point to the presence of a rapidly evolving disturbance in the relationship between regulators for dentistry and dental hygiene in Canada [11, 12]. Accusatory reports citing placation to public fears versus perceived laxity on PPE requirements between these two regulatory bodies are representative of problematic communication on practice standards experienced by OHCPs at this time [11].

To better understand the experience of dental hygienists and the overall impact of new pandemic guidelines on the practice of dental hygiene, a closer analysis of how guidelines were experienced and perceived by practicing dental hygienists warranted further exploration. The objective of this study therefore was to explore via descriptive thematic analysis of qualitative questions posed to dental hygienists over a follow-up period of December 2020 to January 2022, how dental hygienists experienced and perceived: i) dental hygiene practice during the COVID-19 pandemic, and ii) their regulatory body’s COVID-19 guidelines.

## Methods

### Design and settings

This qualitative descriptive (QD) study was nested within a prospective cohort study and used open-ended questions to collect descriptive data from dental hygienists on how they experienced and perceived dental hygiene practice and their regulator’s guidelines during the COVID-19 pandemic. QD is a design widely used within qualitative health care literature and is acknowledged as an appropriate method by which to capture the diverse lived-experiences and insights of individuals in their own words to better understand events or phenomena [13, 14]. QD is also considered most appropriate for use in studies seeking descriptive information that can inform and refine policy or interventions, rendering it a suitable methodology for use in this study [14, 15]. The survey design of this study meant that data collected from eligible participants was self-reported. Self-reported data is a sensitive issue within research, particularly concerning participant bias and validity of the data [13]. However, self-reported data offers many advantages to researchers and the use of open-ended, free-text responses within online survey research has become an acceptable study design and become increasingly attractive to, and mainstream within QD studies [13, 16].

Canada is the second-largest country in the world, and participants were recruited across geographically disperse provinces in Canada. The use of self-reported survey data was determined to be the most appropriate and accessible form of design to achieve a robust sample given the expansive geography, and that the researchers sought to recruit an arrayed sampling of dental hygienists from across the country. Use of self-reported data within a longitudinal web-based study was strategically designed by the researchers to negate challenges in accessing participants to collect data [16]. This study design was also strategic to combat challenges stemming from the COVID-19 pandemic related to inter-provincial travel restrictions and social distancing mandates in Canada at this time, acknowledged by the researchers as a major limitation to accessing participants in an in-person capacity.

### Recruitment and participants

Canadian registered dental hygienists were invited to participate in this study. Eligibility criteria included being registered and licensed to practice dental hygiene in Canada during the study period, and no previous history of COVID-19 infection. Prospective participants were identified through the registers of provincial dental hygiene licensing bodies (BC, AB, MB, ON, QC, NB, NS and NL). Invitations to participate were sent out to 30,444 registered dental hygienists via email through their provincial licensing body. A follow-up invitation was sent to prospective participants 2 weeks following initial invitation and regular reminders were sent until a viable sample size was achieved. Of those invited, 958 consented to participate. Sixty-five participants did not complete the baseline questionnaire, 9 indicated retiring during the study period and 8 reported previous COVID-19 infection and were excluded from the study. A total of 876 participants provided informed consent and were invited to join the longitudinal phase of the study which included a nested QD study of open-ended questions posed to participants at baseline and final follow-up. To combat bias and promote validity in the self-reported data, all participants were anonymous, and the researchers were blinded to participants’ identity. Participant anonymity in the study was designed to capture truthful interpretations of events from participants and allow for freedom of expression in discussing their experiences and perceptions of regulator’s guidelines for practice during the COVID-19 pandemic. Four hundred consented participants elected to participate in the nested qualitative component of this study at baseline and 247 participants participated at final follow-up.

### Data collection

Canadian registered dental hygienists were invited to participate in a longitudinal online survey hosted on the Lime Survey platform, housed on a secure server behind firewall at McGill University. This was administered between December 9, 2020, and January 5, 2022. A 76-question survey was adapted from WHO Unity Study protocols for assessment of COVID-19 risk among health care workers and administered at baseline (December 9, 2020, to January 3, 2021) (Additional file 1) [17]. Demographic survey questions included age, sex, ethnicity, primary practice location, number of practices, and type of practice. An open-ended question was posed to gather information on participant’s concerns regarding dental care during the pandemic. Data were collected on dental hygiene care provided to patients in the previous 2 weeks as well as self-reported SARS-CoV-2 infection status. Upon completion of the baseline questionnaire, six follow-up surveys were administered every 2 months for a 12-month period. Participants were invited to complete the clinical activity survey and provide information on their self-reported SARS-CoV-2 infection and vaccinations statuses. An additional open-ended question was posed to study participants in the final survey (administered November 30, 2021, to January 9, 2022), pertaining to perspectives on their regulatory body’s COVID-19 guidelines (Additional file 2). This study reports only the analysis of the open-ended questions on participants perceptions of providing care during the pandemic and their experiences in interpreting and implementing new guidelines for practice. All questions were vetted by the research team to assess for clarity and appropriateness of the question for the sample population (dental hygienists) and suitability to inform the research questions. Questions were also pilot tested on a subset of consented participants. All participants responses in this study were anonymous to minimize any impact of social desirability bias on the data that arise in discussions of hierarchical power imbalances such as that between dental hygienists and their professional regulatory body. Questionnaires were available in both English and French. Items related to details of clinical activities/dental hygiene care provided to patients, COVID-19 tests, and vaccination were analyzed and reported in a separate paper.

### Preunderstanding

All authors in this study have extensive backgrounds as practicing oral health care providers and researchers in Canada with experience across qualitative and quantitative methodologies. LM and LR are both registered dental hygienists licensed with the College of Dental Hygienists of Nova Scotia and self-identify as female. However, neither practiced clinical dental hygiene in a public setting during the time of this study and were excluded from eligibility.

### Ethical considerations

This study protocol was approved by the McGill University Research Ethics Board (A06-M49-20A (20–06-018)) and Dalhousie University’s Health Sciences Research Ethics Board (REB# 2021–5716). Participants were provided with a detailed outline of the study including author identity, purpose, goals, and their rights as a voluntary participant prior to providing informed consent. Participants maintained the right to withdraw from participation at any time without giving rationale. Informed consent was provided via electronic signature. Anonymity and confidentiality of participants was maintained throughout the data collection process and reporting of study findings.

### Data analysis

A descriptive analysis was performed to report absolute and relative frequencies of respondent characteristics. Analysis of open-ended questions followed a qualitative descriptive methodology using thematic analysis and coding. This analysis concentrated on self-reported data provided in response to two open-ended questions posed to participants at subjectively different time points in the study; the first question was posed at baseline (December, 2020) and the second at the final follow-up timepoint (January, 2022). A full 12-months of the pandemic experience separated the data sets. An inductive approach was used to analyze the data, as the authors sought to use participants’ responses to generate new understanding of how new guidelines were experienced and perceived by dental hygienists. Thematic analysis involved assignment of each data set to two qualified members of the research team for theme identification. LM and LR led the thematic analysis and reviewed all responses to both open-ended questions in their entirety. Thematic identification was conducted by extracting participant responses from the LimeSurvey questionnaire and insertion of all quotes into an electronic thematic spreadsheet. No themes were predetermined and data was primarily organized based on novel major emergent themes and subsequently subjected to further analysis for thematic development and categorization into sub-themes (LM and LR). Next, the initial thematic categories and sub-categories identified were shared with all authors and analyzed again against participant responses. Trustworthiness in the interpretation of the data was achieved through re-reading participant responses and group discussion across multiple meetings to confirm most prominent emergent themes. The authors used these discussions to identify and rectify personal biases in interpretation and engage reflexively with the data in order to achieve consensus on themes and subthemes that most accurately reflected participant experiences and perceptions. Analysis was conducted until theme saturation was achieved in the data and no new ideas were revealed. Thematic summary tables were next constructed as method to organize the data into major theme and sub-themes identified. Dominant quotes that exemplified and strengthened the researchers’ interpretation of each sub-theme were identified for inclusion within the results. Attentiveness to the COREQ checklist criteria for reporting qualitative research was observed by all authors in both study design and analysis of the data (Additional file 3) [18].

## Results

Of 1530 dental hygienists who volunteered to participate, 958 provided informed consent and 893 completed the survey for a completion rate of 93.2%. Participants who did not meet the inclusion criteria were excluded (*n* = 17); a total of 876 participants were invited to participate in the longitudinal phase of the study. Respondents were primarily female (97.8%), Caucasian (86.1%), with a median age of 42 years (IQR 19 years). The majority of respondents were from British Columbia, Alberta and Ontario (26.3, 25.3 and 24.0% respectively), followed by Quebec (9.6%) and Manitoba (9.2%). Of respondents, 92.7% reported working primarily in clinical dental hygiene alongside a dentist in private/public sectors. The majority worked in an urban setting (86.4%) and practiced in only one office (78.3%). A summary of the sociodemographic responses of the participants is outlined in Table 1.

**Table 1:** Characteristics of respondents at baseline

		Total ***n*** = 876 (%)
**Age**	years (median (IQR))	42 (19)
**Sex**		
	Female	857 (98.6)
	Male	19 (2.2)
**Ethnicity**		
	White (Caucasian)	754 (86.1)
	Asian	79 (9.0)
	Arab	6 (0.7)
	Black	6 (0.7)
	Indigenous Aboriginal	6 (0.7)
	Latin American	6 (0.7)
	Mixed	11 (1.3)
	Others	98 (0.9)
**Province**		
	Alberta	222 (25.3)
	British Columbia	230 (26.3)
	Manitoba	81 (9.2)
	Ontario	210 (24.0)
	Quebec	84 (9.6)
	New Brunswick	12 (1.4)
	Nova Scotia	26 (3.0)
	Newfoundland and Labrador	11 (1.3)
**Type of Community Served**		
	Urban	757 (86.4)
	Rural	115 (13.1)
	Remote	4 (0.5)
**Number of practices**		
	1	686 (78.3)
	2	150 (17.1)
	3	30 (3.4)
	> 3	10 (1.1)
**Type of Practice**		
	Clinical dental hygiene	812 (92.7)
	Independent dental hygiene	25 (2.9)
	Other	39 (4.5)

### Observations on pandemic dental care provision (Q1)

At baseline, the question, “Please provide any observations you have concerning dental care provision during the COVID-19 pandemic” was posed to participants. Four hundred participants provided a response to this question. Three major themes were identified as they related to workplace compliance, patient treatment and communication of practice protocols/guidelines. A fourth theme of ‘Other’ was included to categorize responses that fell outside of dominant themes. Following major theme identification, all participant responses were further sub-coded and categorized based on emergent sub-themes in the data. Table 2 provides a summary of major and sub-themes identified from this question.

**Table 2:** Summary of major themes of open-ended question 1 (baseline)

“Please provide any observations you have concerning the dental care provision during the COVID-19 pandemic”			
Major Themes			
Workplace Compliance	Patient Treatment	Communication of Practice Guidelines & Protocols	Other
Sub-themes			
• PPE/equipment/ settings • Positive patient/ workplace compliance • Employer directives • Co-worker behaviour • Conflicting opinions/ office tension	• Personal anxiety/ fear of becoming ill • Patient/office screening processes • Fearful or uncompliant patients • Refusal to treat	• Discrepancies between dental hygiene/dentistry regulatory bodies • Dissatisfaction with regulatory body communication • Differences in regulation and protocol province-province • Discrepancies between regulatory body and public health recommendations • Satisfaction with regulatory body communication	• General anxiety/ fear of pandemic situation • Mental and physical health toll • Contradictory scientific evidence • Non-applicable

The majority of participants used this opportunity to offer observations related to compliance of their workplaces with guidelines implemented in response to the COVID-19 pandemic. Other dominant themes centered on observations relating to patient treatment, screening practices and the communication of practice regulations and protocols. Identified in participant responses, initial perceptions of the implementation and communication of new practice guidelines was contributary to sentiments of concern with the daily operations of their workplace environments, including anxiety and ill-preparedness. Participants also reported strains on interpersonal relationships with coworkers and employers, attributed to differences in interpretation of pandemic severity and safe practices.

#### Challenges within employment and practice settings

Anxiety and frustration surrounding resuming clinical practice was commonly cited by participants in relation to the capacity of practice settings to implement recommended modifications and have appropriate provisions in place to be compliant with guidelines. Participants indicating concerns with equipment, the clinical setting and sourcing PPE noted,

*“The lack of use of N95 masks. Even though there are AGP happening in the next room and the rooms are not separated by a wall.”*

*“I still have concerns regarding air exchange rates and aerosal settling times and how long we need to leave a room before we can provide treatment in it on another patient. [..]The difficulty sourcing approved N95 masks has also been difficult.”*

Concerns about workplace setting were also noted as extending beyond the operatory to challenges in adhering to social distancing and masking guidelines for communal work and staff gathering places.

*“I eat my lunch in my car (back seat covered with washable towels). My main concern is the lunch room--despite past efforts in staggering lunch hours, it is inevitable to share the space with a co-workers. As per my observations people appear to let their guards down during this particular time of day.”*

The ill-compliance of co-workers in adhering to regulatory guidelines was also a recurrent theme across participant responses and commonly identified as contributing to other themes such as personal anxiety/fear of becoming ill and office tension. As several participants noted,

*“not all staff members are as concerned about PPE or cross contamination as I am....so not all staff members wear their masks correctly or all the time [ …*] *it concerns me that the dentists and assistants don’t change their PPE gowns after each patient.”*

*“The infection control is not consistent in the clinic as people have many differing translations of what is required and when”.*

#### Conflicting messaging from regulators

An issue raised by participants related specifically to the dissemination and communication of new guidelines. Under this theme were the implications of inconsistent guideline messaging and their translation to patient care. Conflicts between dentistry and dental hygiene regulators were identified as a source of frustration for dental hygienists striving to be compliant with guidelines. As this participated expressed,

*“There is such confusion in what is ethically needed/acceptable to preform in order to keep everyone safe. Why [aren’t] the governed bodies on board with the same protocols?? Aren’t we all dealing with the same virus?? Why should one be less attentive to the protocols than the other??”*

This data concurrently identified the weight of inconsistent messaging on dental hygienists’ personal and professional satisfaction in their position. Participants cited struggles they faced in attempting to be compliant with their college’s practice guidelines while also adhering to employer directives.

*“dentists seem to be more concerned with having full schedules then they are with their staff’s safety. At this point in the pandemic, I do not feel safe being forced to wear a KN95 all day through multiple clients.”*

Participants who expressed frustration related to conflicting regulations often identified their province of practice. Discontent with guidelines was identified geographically based on the epidemiological trends at that timepoint within provinces. The data suggests that geographically across Canada, the perception of dental hygienists on their regulator’s decision-making for guidelines and implementation was influenced by provincial factors. As such, there may exist differing interpretations of the lived experience of pandemic regulations for dental hygienists across Canada at this time.

### Perceptions on regulatory body guidelines (Q2)

A second open-ended question was posed to participants at the final follow-up timepoint (November 30, 2021, to January 9, 2022) asking, “What is your perspective on your regulatory body’s COVID-19 guidelines?” Collected 12-months into this study, this placed the majority of participants as having worked under new guidelines for dental hygiene practice for more than 1 year, based on re-opening timelines across provinces [3]. Identical processes for coding and thematic categorization used for Q1 were applied to the analysis of this question (Q2). A total of 247 participants offered a response to this question. Thematic analysis revealed three major themes: evaluation of satisfaction, inconsistent messaging and provincial practices and messaging. Table 3 provides a summary of major and sub-themes identified from this question.

**Table 3:** Summary of major themes of open-ended question 2 (follow-up)

“What is your perspective on your regulatory body’s COVID-19 guidelines?”		
Major Themes		
Evaluation of Messaging	Inconsistent Messaging	Provincial Practices and Messaging
Sub-themes		
• Satisfied/Acceptable • Poor/Confusing/Non-Direct • Requiring clearer directives on vaccination • Too much/overwhelming • No opinion	• Discrepancies between dental hygiene/ dentistry regulatory bodies • Messaging inconsistent with emerging/available scientific evidence • Discrepancies between provincial regulatory bodies and public health	• Discrepancies province-province • Desire for Pan-Canadian guidelines

#### Evolution of guideline satisfaction

Noteworthy in the results, majority response rate fell within the category of ‘evaluation of messaging’. Sub-theme analysis revealed that at this point in their pandemic experience, the majority of participants expressed satisfaction or indicated that messaging from regulators on guidelines for practice were acceptable. These responses reveal a noted evolution in participants’ personal evaluation of their college’s regulatory guidelines since baseline.

*“They’ve been doing all they can to help guide us during these trying times”.*

*“They are thorough and adhere to the provincial health officers direction”.*

Satisfaction was also evaluated by participants in relation to communication practices. Positive perceptions of dental hygiene regulators in communicating and disseminating timely guideline updates over the course of the pandemic’s evolution were evidenced in responses,

*“I thought they were efficient with their information in terms of time and gave complete information”.*

*“I am happy with the guidelines and [they] have room to adopt or change as we move forward with more information”.*

#### Prevailing inconsistencies

While the data suggests a positive shift in participants overall perspective on regulator’s guidelines, closer analysis of responses within this category noted lingering grievances pertaining to inconsistent messaging between dental hygiene and dentistry regulators.

*“I am happy with them but wish they would be a little more detailed and correlate with CDA or ADA better.”*

*“CRDHA guidelines are excellent but often come into conflict with the ADA. The ADA doesn’t seem to want to include all the PPE/protocols that my association does.”*

*“I think they did a great job, but I wish they would have worked together with the DDS college so that the guidelines were the same from day 1.”*

Inconsistencies in messaging continued to be identified as problematic to compliant adherence to guidelines by dental hygienists. This was also inclusive of messaging described as indirect or utilized verbiage of ‘recommendation’ versus prescribed regulation; contributing to confusion for application into practice. One participant offered,

*“They were confusing and up to interpretation at the beginning and then never clarified. Most practices figured out their regulations by discussing with colleagues instead of the regulatory body”.*

This excerpt, amongst others identifies the impact of communication breakdowns and ambiguous terminology on perceived trust between registrants and their regulatory bodies.

#### Sentiments of safety

The data suggests that for some dental hygienists, although inconsistencies prevailed between dental hygiene and dentistry regulators, the evolution of practice protocols over the months of the pandemic worked to bridge gaps in recommendations and messaging to arrive at a satisfactory interpretation of the guidelines. This change in perception can be attributed in part to the low exposure and infections rates noted within the dental practice setting; sentiments alluded to in the data as contributing to an increased comfort in providing dental hygiene services during this time [9, 10].

*“Appropriate - science based and change according to changes in community case levels”.*

*“The guidelines are comprehensive and protects dental hygienists in having a safe work environment”.*

*“I am very happy with their guidelines. By staying at a higher level of concern and restrictions allows me to continue seeing all patients with no vaccine barriers.”*

## Discussion

The data derived from these two open-ended questions provides new and corroborative interpretations of the concerns and challenges faced by dental hygienists in navigating the COVID-19 pandemic in Canada. Concerns such as anxiety/fear of becoming infected and general feelings of uncertainty regarding the safety of oral care provision at this time have been reported in the limited literature available specific to dental hygienists [6, 19–21]. Elsewhere, similar concerns and anxieties amongst OHCPs overall have been widely reported regarding virus transmission, PPE, use of AGPs and personal risk of infection during oral care delivery during the pandemic [7, 22–25].

Particular to our study, the findings provide valuable insight to how new practice guidelines and perception of regulator’s messaging was perceived and evolved over the course of the pandemic. It must be noted however, that these two opened-ended questions posed to dental hygienists pertained to overall observations on the pandemic experience in December, 2020 and then specifically about their perception of regulatory guidelines 12 months later, in January, 2022. As a result, the vast differences in subject matter and timepoint between questions threatens the validity of inferential interpretations from the data. However, responses provided by dental hygienists about their COVID-19 experience and perception of new guidelines offer new insight on the strengths and weaknesses of how the profession and dental hygiene care was regulated during this time.

Some pandemic concerns shared by participants at the time of Q1 (baseline) were recognized to have been partially mitigated by the time of Q2 (final follow-up). Concerns regarding transmission of the virus and the risk to dental hygienists in the dental office were interpreted to be subdued in part due to shifting epidemiologic patterns, increased scientific evidence on transmission, and the demonstrated success of public protection measures [9, 26]. Concerns specific to the perceived competency of dental hygiene regulatory colleges to protect the health and safety of dental hygienists and patients during the pandemic were meanwhile noted as linked to perceived consistency, clarity and authoritative stance of guideline messaging. These qualities were found to have significant bearing on participant’s perceived trust in their college to effectively regulate the profession under pandemic conditions [27, 28]. A reliance on word of mouth, social networks within the profession, and non-scholarly sources were cited by participants both in this study and elsewhere as more accessible sources of information gathering on pandemic guidelines [6, 25, 29].

It is important to note that these challenges are not novel to the field of dental hygiene. Participants expressing dissatisfaction with the current guidelines illuminated prevailing challenges faced by many health profession agencies and policy-makers at this time [27, 28]. For those charged with communicating health information during heightened epidemiological crises, there is an identified responsibility to acknowledge temporality in what is currently known as evidence emerges and changes [30, 31]. Feelings of uncertainty within disease-related events are found to be exacerbated by ambiguous/incomplete information, complexity of information presented, and perceived unpredictability of disease trends [30].

Inconsistent guidelines on PPE/equipment and practice setting standards were highly cited by participants in this study. Studies examining dentists’ knowledge of PPE recommendations have demonstrated a prevailing lack of consensus on proper usage, suggesting that inconsistent messaging on best practice protocols permeate throughout the oral health professions locally and internationally [32, 33]. Inconsistent messaging on clinical recommendations between regulators for dentistry and dental hygiene within a singular province were identified as a source of stress. The prevalence at which this theme emerged was surprising given that it is anticipated regulators were working from the same scientific body of evidence to inform guidelines recommendations [1]. Emergent research on effective health information communication during the COVID-19 pandemic points to the importance of limiting the number of spokespersons and consistency in messaging to promote compliance [30].

In Canada, reports from the Chief Dental Officer on evidence to support the safe return to practice were readily available and regularly updated as a resource to guide provincial regulators [1]. Despite resources available, the discrepancies raised by participants in this study draws attention to the stark differences in approach to regulating oral health services regionally across Canada during the pandemic. While many provincial regulators assumed responsibility for guideline creation for their respective profession, elsewhere more collaborative approaches were documented. In Nova Scotia (NS), inconsistent messaging was proactively mitigated by a mandated requirement from the province’s Chief Medical Officer for collaboration from all four of the province’s registered OHCPs. The creation of a provincial collaborative for the oral health professions was formed to establish consensus on scientific evidence and creation of a single set of guidelines to direct all OHCPs in NS [2]. Guidelines were regularly updated by the collaborative as new evidence emerged and prompt dissemination was facilitated by each regulatory college to their registrants [2]. The success of this collaboration is consistent with best practice recommendations for improving health policy messaging cited in the literature [27, 30, 31, 34]. Corroborated by responses of participants in our study, there is an identified need for increased collaboration amongst oral health regulators. The formation of working groups and collaborative networks are viable solutions for creating guidelines for practice that are consistent, direct and promote streamlined approaches to oral health care in pandemic contexts and beyond [2, 34].

Within the registrant-regulator relationship, the breakdown of trust is compounded in situations where there is perceived discontent on regulator’s initial response to a crisis or where pre-existing trust issues exist [35]. Compounding this effect is that responsibility for health care services in Canada is assumed by independent provinces [26]. This can be attributed to differences in evidence interpretation and decision-making with reverberating effects on the decisions of regulators for practice guidelines [26]. What the data reveal however, is that the erosion of trust between regulators and registrants can be incrementally repaired through increased transparency regarding rationale, decision-making and demonstrated willingness to collaborate with other stakeholders to produce best practice protocols [27, 30].

## Limitations

A noted limitation to this study is that the two open-ended questions spoke to concerns expressed by dental hygienists at two very different timepoints in the study (baseline and final follow up). In addition, Q1 asked for observations on overall pandemic experience while Q2 was specific to perceptions of regulatory guidelines. The considerable differences in timepoint and subject matter of these two questions is therefore limiting to the researchers’ ability to substantiate related conclusions made from two subjectively different data sets. Future analyses would be strengthened by separate cross-sectional analyses of these questions from study commencement to completion.

Further to this, the sample population was restricted to hygienists in Canada. The geographic context, evolution of the COVID-19 virus and emergence of new evidence has implications on regulators’ ongoing approaches to guidelines. This also has implications on how dental hygienists perceive and apply them to practice. Participants in this study were also self-selected and volunteer bias may have impacted the results. Further, the authors acknowledge that this study relied on self-reported data and despite strategies employed, there maintains a risk of bias that is a limitation to the findings.

## Future research

As the pandemic and emergence of new variants evolves, the long-term impacts of the COVID-19 on dental hygienists and provision of services remains to be fully realized [5]. This study has identified that further research is warranted on how dental hygienists have continued to adapt to regulatory guidelines communicated locally and nationally and the precedence of these guidelines in establishing a ‘new normal’ for dental hygiene care in Canada. This evolving gap also suggests that there exists merit in further exploration of the efficacy of interprofessional working groups to devise guidelines for oral health care nationally and globally.

## Conclusions

Dental hygienists in this study reported challenges to dental hygiene care provision during the COVID-19 pandemic that included low PPE, co-worker, and practice setting compliance with new guidelines for minimizing risk of disease. These stressors were cited as inherently linked to conflicting guideline messaging from regulatory bodies for OHCPs, contributing to frustration and anxiety experienced by dental hygienists within the profession and interprofessionally. Dental hygiene regulators have distinct obligations to direct the practice of dental hygiene care to be compliant with current evidence and transparent in their messaging. This study highlights that inconsistent and conflicting messaging within practice guidelines disseminated by oral health regulators during the pandemic reflect a need for increased collaboration amongst OHCPs to facilitate streamlined protocols for patient care within pandemic disease contexts at the provincial and pan-Canadian level.

## Supplementary Information

**Additional file 1.** Baseline Questionnaire. Description: Baseline questionnaire distributed to participants including open-ended question 1.

**Additional file 2.** Follow-Up Questionnaire. Description: Follow-up questionnaire distributed to participants including open-ended question 2.

**Additional file 3.** COREQ (Consolidated Criteria for Reporting Qualitative Research) 32-Item Checklist.

## Data Availability

The datasets used and analyzed during the current study are available from the corresponding author on reasonable request.

## Abbreviations

SARS-CoV-2: Severe Acute Respiratory Syndrome Coronavirus 2
PPE: Personal Protective Equipment
AGP: Aerosol-Generating Procedures
BC: British Columbia
AB: Alberta
SK: Saskatchewan
MB: Manitoba
ON: Ontario
QC: Quebec
NB: New Brunswick
NS: Nova Scotia
NL: Newfoundland & Labrador
WHO: World Health Organization
RDSO: Royal College of Dental Surgeons of Ontario
CDHO: College of Dental Hygienists of Ontario
CDA: Canadian Dental Association
CRDHA: College of Registered Dental Hygienists of Alberta
ADA: American Dental Association
DDS: Doctor of Dental Surgery
OHCP: Oral Health Care Provider
