# Supplementary material for: Canadian dental hygienists’ experiences and perceptions of regulatory guidelines during the COVID-19 pandemic: a qualitative descriptive analysis
Source: BMC Health Serv Res. 2022 Dec 22;22:1570. doi: 10.1186/s12913-022-08925-z (PMC9773656; doi:10.1186/s12913-022-08925-z)
Supplement: Supplementary file 1 — Additional file 1. Baseline Questionnaire. Description: Baseline questionnaire distributed to participants including open-ended question 1. [file 12913_2022_8925_MOESM1_ESM.docx]

**COVID-19 incidence rates among
Canadian dentists and dental hygienists: a cohort study**

**(Updated: 28/10/2020)**

**Baseline and Follow-up Questionnaires**

**Sections 5, 6, 7, 8 will be repeated for all follow-up visits. Section 10 is specific to the qualitative nested study.**

*Questions with an asterisk (*) are mandatory.*

Contents

[Section 1. Contact information 3](#_Toc54813686)

[Section 2. Demographics & Comorbidity 4](#_Toc54813687)

[Section 3: Professional Information 7](#_Toc54813688)

[Section 4: Potential for exposure 9](#_Toc54813689)

[Section 5: COVID-19 Tests and symptoms 11](#_Toc54813690)

[Section 6: Activities 15](#_Toc54813691)

[Section 7: In-person dental care episodes 16](#_Toc54813692)

[Section 8: Co-workers 19](#_Toc54813693)

[Section 9: COVID-19 Anxiety 21](#_Toc54813694)

[Section 10: Comments 22](#_Toc54813695)

# Section 1. Contact information

**The contact information you provide, on this page, will be kept**

**confidential and will only be used for the purpose of communicating**

**matters pertinent to this study.**

1. **Please enter your contact information** *

Please write your answer(s) here:

First name: ________________________________

Second name / Surname: _____________________

Email: _____________________

Phone: ____________________________________

Please **do not** use country code or leave spaces for your phone number.

Example: **5141238888**

# Section 2. Demographics & Comorbidity

1. **How old are you:** *

Your answer must be between 18 and 99

Only an integer value may be entered in this field.

Please write your answer here: __________years

1. **Sex:** *

Choose one of the following answers

Please choose **only one** of the following:

- Female
- Male

1. **Gender**

Check all that apply

Please choose **all** that apply:

- Agender
- Genderqueer
- Gender fluid
- Man
- Non-binary
- Questioning or unsure
- Transgender
- Trans man
- Trans woman
- Woman
- Prefer not to disclose
- Other: ______________

1. **Please indicate below which group best describes you:** *

Choose one of the following answers

Please choose **only one** of the following:

- White (Caucasian)
- South Asian (e.g., East Indian, Pakistani, Sri Lankan, etc.)
- Chinese
- Black
- Filipino
- Latin American
- Arab
- Southeast Asian (e.g., Vietnamese, Cambodian, Laotian, Thai, etc.)
- West Asian (e.g., Iranian, Afghan, etc.)
- Korean
- Japanese
- Aboriginal
- Other

1. **Smoker**

Please choose **only one** of the following:

- Yes
- No

1. **Have you ever had following disease(s)/condition(s)?** *

Please choose the appropriate response for each item: (Yes/No/Unknown):

|  | Yes | No | Unknown |
| --- | --- | --- | --- |
| Obesity |  |  |  |
| Cancer |  |  |  |
| Diabetes |  |  |  |
| HIV/other immune deficiency |  |  |  |
| Asthma (requiring medication) |  |  |  |
| Chronic lung disease (non-asthma) |  |  |  |
| Chronic liver disease |  |  |  |
| Chronic haematological disorder |  |  |  |
| Chronic kidney disease |  |  |  |
| Chronic neurological impairment/disease |  |  |  |
| Organ or bone marrow replacement |  |  |  |
| Heart condition |  |  |  |

1. **Any other comorbidity:**

Please write your answer here: __________________________________________________________

1. **Are you currently pregnant?** *

Please choose **only one** of the following:

- Yes
- No
- Unknown

1. **Specify trimester:**

**Only answer this question if the following conditions are met:**

*Answer was 'Yes' at question 10 (Are you currently pregnant?)*

Please choose **only one** of the following:

- First trimester
- Second trimester
- Third trimester

1. **What is the estimated delivery date?**

**Only answer this question if the following conditions are met:**

Answer was ' Yes' at question 10 (Are you currently pregnant?)

Answer must be greater or equal to today

Please enter a date:

# Section 3: Professional Information

1. **Please indicate the province where your primary practice, as a dentist or a dental hygienist, is located:**

*(office at which you work the most during a week)* *

Please choose **only one** of the following:

- Alberta
- British Columbia
- Manitoba
- New Brunswick
- Newfoundland and Labrador
- Nova Scotia
- Ontario
- Prince Edward Island
- Quebec
- Saskatchewan
- Northwest Territories
- Nunavut
- Yukon

1. **Please indicate the postal code of your primary practice as a dentist or a dental hygienist (office at which you work the most during a week):** *

Please enter a valid postal code (E.g.,A1F3Y7)

Please write your answer here:

1. **In your primary practice as a dentist or a dental hygienist, are you largely serving a:**

Choose one of the following answers

Please choose **only one** of the following:

- Metropolitan community
- Urban community
- Suburban community
- Rural community
- Remote community

1. **How many offices do you work in each week?**

Please choose **only one** of the following:

- One per week
- Two per week
- Three per week
- More than three per week

1. **Are you a dentist or a dental hygienist?** *

Please choose **only one** of the following:

- Dentist – General practitioner
- Dentist – Speciality practitioner
- Dental Hygienist
- I have retired from clinical practice since:_____________(Date)

1. **Please specify your speciality** *

**Only answer this question if the following conditions are met:**

Answer was 'Dentist-Specialist practitioner' at question 17

Please choose **all** that apply:

- Dental Public Health
- Endodontics
- Oral and Maxillofacial surgery
- Pediatric Dentistry
- Oral Medicine and Pathology
- Periodontics
- Oral and Maxillofacial Radiology
- Prosthodontics
- Orthodontics and Dentofacial Orthopedics
- Other: _____________________________

1. **Please specify your practice type as a dental hygienist.**

- Clinical Dental Hygienist *(Working alongside a dentist in private or public sectors)*
- Independent Dental Hygienist *(Working independently or along with other dental hygienists, but not with a dentist, in private or public sectors)*
- Other:_______________________________

1. **Is the clinic where you provided care most of the time over the past 2 weeks:** *

Please choose **only one** of the following:

- Open [no walls between dental chairs]
- Semi-open [some areas are open to each other while others have walls or other barriers separating them]
- Closed concept [all areas are separated by walls]
- Other

# Section 4: Potential for exposure

1. **Have you travelled outside Canada, or have you returned to Canada, in the past**

**28 days?** *

Please choose **only one** of the following:

- Yes
- No

1. **In past 28 days, have you travelled within or outside your province or region of residence?**

- Yes
- No

1. **If yes, please specify the following:**

- Travelled only with in the region of my current residence
- Travelled outside the region, but within the province of my current residence
- Travelled outside the province of my current residence

1. **Have you shared a living space with someone (family or other), in past 28 days? ***

Please choose **only one** of the following:

- Yes
- No

1. **Did any of your co-habitants attended primary or secondary school in-person, in the past 28 days?**

- Yes
- No

1. **Did any of your co-habitants attend a day care in-person during the past 28 days?**

- Yes
- No

1. **Has anyone whom you are living with had a positive test for COVID-19, in the past 28 days?** *

Please choose **only one** of the following:

- Yes
- No
- Unknown

1. **Has anyone whom you are living with had any symptoms that made you suspect they have COVID-19, in the past 28 days?** *

Please choose **only one** of the following:

- Yes
- No
- Unknown

1. **In past 28 days, have you attended a health care facility (other than the clinics you provide care) for yourself or a companion?**

- Yes
- No

1. **In past 28 days, have you attended any private gatherings with persons outside your household?**

- Yes
- No

1. **In past 28 days, have you attended any public gatherings/events with 50 or more people?**

- Yes
- No

1. **Have you ever worked at a facility which cares for COVID-19 patients?** *

Please choose **only one** of the following:

- Yes
- No

1. **Have you ever provided any form of dental care for patients with COVID-19?***

Choose one of the following answers

Please choose **only one** of the following:

- Yes
- No
- Unknown

# Section 5: COVID-19 Tests and symptoms

1. **Have you been tested for COVID-19, other than this project since the last follow-up survey?**

- Yes
- No

1. **Please specify the type of test:**

- Nasopharyngeal swab sample and PCR based test
- Nasopharyngeal swab sample and antigen test
- Saliva sample (Other than the test performed in this project) and PCR based Test
- Saliva sample (Other than the test performed in this project) and antigent Test
- Serum sample (Blood) and antibody testing
- Other:______________

1. **Date of testing:**________________
2. **Were you tested positive for SARS-COV2 or COVID-19 in this test?**

- Yes
- No
- Inconclusive
- Still waiting for the results

1. **Did you ever test positive for COVID-19?** *

Please choose **only one** of the following:

- Yes
- No

1. **If yes, date of testing:** *

Answer must be less or equal to ‘today’

Please enter a date:

1. **Have you experienced any respiratory symptoms (e.g., sore throat, cough, running nose, shortness of breath) of COVID-19, in last 28 days?** *

Please choose **only one** of the following:

- Yes
- No

1. **Date of first symptom onset:**

**Only answer this question if the following conditions are met:**

Answer was 'Yes' at question 40 (Have you experienced any respiratory symptoms (e.g., sore throat, cough, running nose, shortness of breath) of COVID-19?)

Answer must be less or equal to ‘today’

Please enter a date:

1. **Fever (≥38 °C) or history of fever** *

Choose one of the following answers

Please choose **only one** of the following:

- Yes
- No
- Unknown

1. **Date of onset of fever:**

**Only answer this question if the following conditions are met:**

Answer was ' Yes' at question 42 (Fever (≥38 °C) or history of fever)

Answer must be less or equal to ‘today’

Please enter a date:

1. **Sore throat** *

**Only answer this question if the following conditions are met:**

Answer was 'Yes' at question 40 (Have you experienced any respiratory symptoms (e.g., sore throat, cough, running nose, shortness of breath) of COVID-19?)

Choose one of the following answers

Please choose **only one** of the following:

- Yes
- No
- Unknown

1. **Date of onset of sore throat:**

**Only answer this question if the following conditions are met:**

Answer was ' Yes' at question 44 (Sore throat)

Answer must be less or equal to ‘today’

Please enter a date:

1. **Cough** *

**Only answer this question if the following conditions are met:**

Answer was 'Yes' at question 40 (Have you experienced any respiratory symptoms (e.g., sore throat, cough, running nose, shortness of breath) of COVID-19?)

Choose one of the following answers

Please choose **only one** of the following:

- Yes
- No
- Unknown

1. **Date of onset of cough:**

**Only answer this question if the following conditions are met:**

Answer was ' Yes' at question 46 (Cough)

Answer must be less or equal to ‘today’

Please enter a date:

1. **Runny nose** *

**Only answer this question if the following conditions are met:**

Answer was 'Yes' at question 40 (Have you experienced any respiratory symptoms (e.g., sore throat, cough, running nose, shortness of breath) of COVID-19?)

Choose one of the following answers

Please choose **only one** of the following:

- Yes
- No
- Unknown

1. **Date of onset of runny nose:**

**Only answer this question if the following conditions are met:**

Answer was ' Yes' at question 48 (Runny nose)

Answer must be less or equal to ‘today’

Please enter a date:

1. **Shortness of breath** *

**Only answer this question if the following conditions are met:**

Answer was 'Yes' at question 40 (Have you experienced any respiratory symptoms (e.g., sore throat, cough, running nose, shortness of breath) of COVID-19?)

Choose one of the following answers

Please choose **only one** of the following:

- Yes
- No
- Unknown

1. **Date of onset of shortness of breath:**

**Only answer this question if the following conditions are met:**

Answer was ' Yes' at question 50 (Shortness of breath)

Answer must be less or equal to ‘today’

Please enter a date:

1. **Other symptoms** *

Please choose the appropriate response for each item:

|  | Yes | No | Unknown |
| --- | --- | --- | --- |
| Chills |  |  |  |
| Vomiting |  |  |  |
| Nausea |  |  |  |
| Diarrhoea |  |  |  |
| Headache |  |  |  |
| Rash |  |  |  |
| Conjunctivitis |  |  |  |
| Muscle aches |  |  |  |
| Joint aches |  |  |  |
| Nosebleed |  |  |  |
| Fatigue |  |  |  |
| General malaise |  |  |  |
| Loss of appetite |  |  |  |
| Loss of smell /altered sense of smell |  |  |  |
| Loss of taste / altered sense of taste |  |  |  |

1. **Any other symptoms** *

Choose one of the following answers

Please choose **only one** of the following:

- Yes (Please specify below)
- No
- Unknown

Make a comment on your choice here:

1. **Have you stopped working/practicing (even temporarily), in the past 28 days?** *

Please choose **only one** of the following:

- Yes
- No

1. **Please specify your last date of working/practicing:** *

Answer must be less or equal to ‘today’

Please enter a date:

# Section 6: Activities

**These questions are about your clinical activities in the 2 weeks prior to your last working day, or of 2 weeks prior to your COVID-19 positive test; depending on the answer to questions in the previous section.**

1. **During this period, did you spend most of your time at home?** *

Please choose **only one** of the following:

- Yes
- No

1. **During this period, how many times did you leave your home?**

Choose one of the following answers

Please choose **only one** of the following:

- Never
- Once
- Twice
- 3 to 5 times
- 6 to 10 times
- More than 10 times

1. **Please choose the outdoor activities you engaged in during this period:**

(Choose all that applies)

- Shopping (Including shopping for groceries)
- Physical activity in groups (e.g., Gym, sports, dancing)
- Wellness or lifestyle services (e.g., Spa, Hair or Nail Saloons)
- Accompanying family members to events or appointments
- Visiting family or friends in residence or long-term care facilities
- Other:__________________________

1. **During this period did you provide any form of in-person dental care (including**

**consultations)?** *

Please choose **only one** of the following:

- Yes
- No

# Section 7: In-person dental care episodes

**This section refers to the in-person care you provided during the 2 weeks prior to your last working day, or of 2 weeks prior to your COVID-19 positive test; depending on the answer to questions in the previous section.**

1. **During this period how many patients did you provide some form of in-person dental care per day on average?** *

Your answer must be at least 1

Only an integer value may be entered in this field.

Please write your answer here: _________________

Please enter an average number.

1. **During this period how many patients per day required an aerosol-generating procedure?** *

Only an integer value may be entered in this field.

Please write your answer here: _________________

Please enter an average number. If none, enter "0".

1. **During this period did you provide any in-person dental care for COVID-19 positive**

**patients?** *

Please choose **only one** of the following:

- Yes
- No

1. **If yes, for how many COVID-19 positive patients?** *

Your answer must be at least 1

Only an integer value may be entered in this field.

Please write your answer here: _________________

1. **During this period did any of the patients you cared for, have any symptoms that made you suspect they are infected with COVID-19?** *

Please choose **only one** of the following:

- Yes
- No

1. **If yes, how many patients?** *

Your answer must be at least 1

Only an integer value may be entered in this field.

Please write your answer here: _________________

1. **Please specify the types of in-person dental care you provided during this period**

Check all that apply

Please choose **all** that apply:

- Advice and education only
- Tooth extraction
- Radiographs
- Examination and evaluation
- Scaling with hand instruments
- Scaling with ultrasonic scaler
- Abscess drainage
- Mineralized tissue removal with handpiece
- Adjustment of prosthesis or orthodontic appliance
- Pulp removal
- Provision of a prescription for a painkiller
- Provision of a prescription for an antibiotic
- Provision of a prescription for another medication
- Other: ___________________________________

# Section 8: Co-workers

**The questions on this page are referring to the period of 2 weeks prior to your last working day, or of 2 weeks prior to your COVID-19 positive test; depending on the answer to questions in the COVID-19 test and symptoms section.**

1. **During this period how many members of staff** (including dentists, receptionists, dental hygienists, dental assistants and others) **were working with you in the same clinic where you worked most of the time?** *

Your answer must be at least 0

Only an integer value may be entered in this field.

Please write your answer here: _________________

Please enter "0" if none.

1. **During this period did any of your co-workers, at the office you provided care, have a**

**positive test for COVID-19?** *

Choose one of the following answers

Please choose **only one** of the following:

- Yes
- No
- Unknown

1. **Please choose the description(s) that best fit the position of the staff member(s) who had a positive test for COVID-19:** *

Please choose **all** that apply:

- Dentist
- Dental hygienist
- Dental assistant
- Receptionist
- Other:

1. **During this period did any of your co-workers, at the office you provided care, have any symptom which made you suspect that they have COVID-19?** *

Choose one of the following answers

Please choose **only one** of the following:

- Yes
- No
- Unknown

1. **Please choose the description(s) that best fit the position of the staff member(s) who had symptoms similar to COVID-19:** *

Check all that apply

Please choose **all** that apply:

- Dentist
- Dental hygienist
- Dental assistant
- Receptionist
- Other: _________________

# Section 9: COVID-19 Anxiety

1. **Please rate the extent to which each statement applies to you over the last two weeks**.*

|  | Not at all (0) | Rarely, less than a day or two (1) | Several days (2) | More than 7 days (3) | Nearly every day (4) |
| --- | --- | --- | --- | --- | --- |
| - I have avoided using public transport because of the fear of contracting coronavirus (COVID-19) |  |  |  |  |  |
| - I have checked myself for symptoms of coronavirus (COVID-19) |  |  |  |  |  |
| - I have avoided going out to public places (shops, parks) because of the fear of contracting coronavirus (COVID-19) |  |  |  |  |  |
| - I have been concerned about not having adhered strictly to social distancing guidelines for coronavirus (COVID-19) |  |  |  |  |  |
| - I have avoided touching things in public spaces because of the fear of contracting coronavirus (COVID-19). |  |  |  |  |  |
| - I have read about news relating to coronavirus (COVID-19) at the cost of engaging in work. |  |  |  |  |  |
| - I have checked my family members and loved one for the signs of coronavirus (COVID-19). |  |  |  |  |  |
| - I have been paying close attention to others displaying possible symptoms of coronavirus (COVID-19). |  |  |  |  |  |
| - I have imagined what could happen to my family members if they contracted coronavirus (COVID-19). |  |  |  |  |  |
| - I am afraid of getting COVID-19 from a patient or a co-worker |  |  |  |  |  |
| - I am anxious when providing treatment to patients with flu like symptoms |  |  |  |  |  |
| - I fear that the PPE I am using may not be sufficient to protect me against COVID-19 |  |  |  |  |  |

# Section 10: Comments

1. **Please provide any observations you have concerning the dental care provision during the COVID-19 pandemic:**

Please write your answer here:

**Thank you for your participation!**

**You may close your browser now.**
