# Supplementary material for: Canadian dental hygienists’ experiences and perceptions of regulatory guidelines during the COVID-19 pandemic: a qualitative descriptive analysis
Source: BMC Health Serv Res. 2022 Dec 22;22:1570. doi: 10.1186/s12913-022-08925-z (PMC9773656; doi:10.1186/s12913-022-08925-z)
Supplement: Supplementary file 2 — Additional file 2. Follow-Up Questionnaire. Description: Follow-up questionnaire distributed to participants including open-ended question 2. [file 12913_2022_8925_MOESM2_ESM.docx]

**COVID-19 incidence rates among
Canadian dentists: a cohort study**

**Follow-up Questionnaires**

**(Updated 31/8/2021)**

**Sections 7 to 9 are added to the standard follow-up questionnaire (section 1 to 6). Section 8 is specific to the qualitative nested study**

*Questions with an asterisk (*) are mandatory.*

Contents

[Section 1: COVID-19 Vaccine 3](#_Toc81309242)

[Section 2: COVID-19 Tests and symptoms 4](#_Toc81309243)

[Section 3: Activities 9](#_Toc81309244)

[Section 4: In-person dental care episodes 10](#_Toc81309245)

[Section 5: Co-workers 13](#_Toc81309246)

[Section 6: COVID-19 Anxiety 15](#_Toc81309247)

[Section 7: Anxiety due to economic impact of COVID-19 16](#_Toc81309248)

[Section 8: Overall impact of COVID-19 20](#_Toc81309249)

[Section 9: Participant satisfaction 22](#_Toc81309250)

# Section 1: COVID-19 Vaccine

**The following questions are about vaccination against COVID-19**

1. Have you been vaccinated against COVID-19?

(*Answer ‘Yes’ if you have received at least one dose of the COVID-19 vaccine.*

*Note: Certain types of vaccines require more than one dose to protect against COVID-19. You would have been informed at the time of vaccination if you needed a second dose.)*

- Yes
- No

1. How many doses of the COVID-19 vaccine have you received so far?

*Choose one of the following answers*

- One dose
- Two doses
- More than two doses

1. When did you receive your first dose of the COVID-19 vaccine? ____________________
2. When did you receive your second dose of the COVID-19 vaccine?__________________
3. Which vaccine did you receive? *(Choose one of the following answers)*

Was it:

- Pfizer and BioNTech mRNA vaccine
- Moderna mRNA vaccine
- AstraZeneca Oxford vaccine
- Don't know
- Other:

# Section 2: COVID-19 Tests and symptoms

1. **Have you been tested for COVID-19, other than this project since the last follow-up survey?**

- Yes
- No

1. **Please specify the type of test:**

- Nasopharyngeal swab sample and PCR based test
- Nasopharyngeal swab sample and antigen test
- Saliva sample (Other than the test performed in this project) and PCR based Test
- Saliva sample (Other than the test performed in this project) and antigent Test
- Serum sample (Blood) and antibody testing
- Other:______________

1. **Date of testing:**________________
2. **Were you tested positive for SARS-COV2 or COVID-19 in this test?**

- Yes
- No
- Inconclusive
- Still waiting for the results

1. **If yes, date of testing:** *

Answer must be less or equal to ‘today’

Please enter a date:

1. **Have you experienced any respiratory symptoms (e.g., sore throat, cough, running nose, shortness of breath) of COVID-19, in last 28 days?** *

Please choose **only one** of the following:

- Yes
- No

1. **Date of first symptom onset:**

**Only answer this question if the following conditions are met:**

Answer was 'Yes' at question 40 (Have you experienced any respiratory symptoms (e.g., sore throat, cough, running nose, shortness of breath) of COVID-19?)

Answer must be less or equal to ‘today’

Please enter a date:

1. **Fever (≥38 °C) or history of fever** *

Choose one of the following answers

Please choose **only one** of the following:

- Yes
- No
- Unknown

1. **Date of onset of fever:**

**Only answer this question if the following conditions are met:**

Answer was ' Yes' at question 42 (Fever (≥38 °C) or history of fever)

Answer must be less or equal to ‘today’

Please enter a date:

1. **Sore throat** *

**Only answer this question if the following conditions are met:**

Answer was 'Yes' at question 40 (Have you experienced any respiratory symptoms (e.g., sore throat, cough, running nose, shortness of breath) of COVID-19?)

Choose one of the following answers

Please choose **only one** of the following:

- Yes
- No
- Unknown

1. **Date of onset of sore throat:**

**Only answer this question if the following conditions are met:**

Answer was ' Yes' at question 44 (Sore throat)

Answer must be less or equal to ‘today’

Please enter a date:

1. **Cough** *

**Only answer this question if the following conditions are met:**

Answer was 'Yes' at question 40 (Have you experienced any respiratory symptoms (e.g., sore throat, cough, running nose, shortness of breath) of COVID-19?)

Choose one of the following answers

Please choose **only one** of the following:

- Yes
- No
- Unknown

1. **Date of onset of cough:**

**Only answer this question if the following conditions are met:**

Answer was ' Yes' at question 46 (Cough)

Answer must be less or equal to ‘today’

Please enter a date:

1. **Runny nose** *

**Only answer this question if the following conditions are met:**

Answer was 'Yes' at question 40 (Have you experienced any respiratory symptoms (e.g., sore throat, cough, running nose, shortness of breath) of COVID-19?)

Choose one of the following answers

Please choose **only one** of the following:

- Yes
- No
- Unknown

1. **Date of onset of runny nose:**

**Only answer this question if the following conditions are met:**

Answer was ' Yes' at question 48 (Runny nose)

Answer must be less or equal to ‘today’

Please enter a date:

1. **Shortness of breath** *

**Only answer this question if the following conditions are met:**

Answer was 'Yes' at question 40 (Have you experienced any respiratory symptoms (e.g., sore throat, cough, running nose, shortness of breath) of COVID-19?)

Choose one of the following answers

Please choose **only one** of the following:

- Yes
- No
- Unknown

1. **Date of onset of shortness of breath:**

**Only answer this question if the following conditions are met:**

Answer was ' Yes' at question 50 (Shortness of breath)

Answer must be less or equal to ‘today’

Please enter a date:

1. **Other symptoms** *

Please choose the appropriate response for each item:

|  | Yes | No | Unknown |
| --- | --- | --- | --- |
| Chills |  |  |  |
| Vomiting |  |  |  |
| Nausea |  |  |  |
| Diarrhoea |  |  |  |
| Headache |  |  |  |
| Rash |  |  |  |
| Conjunctivitis |  |  |  |
| Muscle aches |  |  |  |
| Joint aches |  |  |  |
| Nosebleed |  |  |  |
| Fatigue |  |  |  |
| General malaise |  |  |  |
| Loss of appetite |  |  |  |
| Loss of smell /altered sense of smell |  |  |  |
| Loss of taste / altered sense of taste |  |  |  |

1. **Any other symptoms** *

Choose one of the following answers

Please choose **only one** of the following:

- Yes (Please specify below)
- No
- Unknown

Make a comment on your choice here:

1. **Have you stopped working/practicing (even temporarily), in the past 28 days?** *

Please choose **only one** of the following:

- Yes
- No

1. **Please specify your last date of working/practicing:** *

Answer must be less or equal to ‘today’

Please enter a date:

# Section 3: Activities

**These questions are about your clinical activities in the 2 weeks prior to your last working day, or of 2 weeks prior to your COVID-19 positive test; depending on the answer to questions in the previous section.**

1. **During this period, did you spend most of your time at home?** *

Please choose **only one** of the following:

- Yes
- No

1. **During this period, how many times did you leave your home?**

Choose one of the following answers

Please choose **only one** of the following:

- Never
- Once
- Twice
- 3 to 5 times
- 6 to 10 times
- More than 10 times

1. **Please choose the outdoor activities you engaged in during this period:**

(Choose all that applies)

- Shopping (Including shopping for groceries)
- Physical activity in groups (e.g., Gym, sports, dancing)
- Wellness or lifestyle services (e.g., Spa, Hair or Nail Saloons)
- Accompanying family members to events or appointments
- Visiting family or friends in residence or long-term care facilities
- Other:__________________________

1. **During this period did you provide any form of in-person dental care (including**

**consultations)?** *

Please choose **only one** of the following:

- Yes
- No

# Section 4: In-person dental care episodes

**This section refers to the in-person care you provided during the 2 weeks prior to your last working day, or of 2 weeks prior to your COVID-19 positive test; depending on the answer to questions in the previous section.**

1. **During this period how many patients did you provide some form of in-person dental care per day on average?** *

Your answer must be at least 1

Only an integer value may be entered in this field.

Please write your answer here: _________________

Please enter an average number.

1. **During this period how many patients per day required an aerosol-generating procedure?** *

Only an integer value may be entered in this field.

Please write your answer here: _________________

Please enter an average number. If none, enter "0".

1. **During this period did you provide any in-person dental care for COVID-19 positive**

**patients?** *

Please choose **only one** of the following:

- Yes
- No

1. **If yes, for how many COVID-19 positive patients?** *

Your answer must be at least 1

Only an integer value may be entered in this field.

Please write your answer here: _________________

1. **During this period did any of the patients you cared for, have any symptoms that made you suspect they are infected with COVID-19?** *

Please choose **only one** of the following:

- Yes
- No

1. **If yes, how many patients?** *

Your answer must be at least 1

Only an integer value may be entered in this field.

Please write your answer here: _________________

1. **Please specify the types of in-person dental care you provided during this period**

Check all that apply

Please choose **all** that apply:

- Advice and education only
- Tooth extraction
- Radiographs
- Examination and evaluation
- Scaling with hand instruments
- Scaling with ultrasonic scaler
- Abscess drainage
- Mineralized tissue removal with handpiece
- Adjustment of prosthesis or orthodontic appliance
- Pulp removal
- Provision of a prescription for a painkiller
- Provision of a prescription for an antibiotic
- Provision of a prescription for another medication
- Other: ___________________________________

# Section 5: Co-workers

**The questions on this page are referring to the period of 2 weeks prior to your last working day, or of 2 weeks prior to your COVID-19 positive test; depending on the answer to questions in the COVID-19 test and symptoms section.**

1. **During this period how many members of staff** (including dentists, receptionists, dental hygienists, dental assistants and others) **were working with you in the same clinic where you worked most of the time?** *

Your answer must be at least 0

Only an integer value may be entered in this field.

Please write your answer here: _________________

Please enter "0" if none.

1. **During this period did any of your co-workers, at the office you provided care, have a**

**positive test for COVID-19?** *

Choose one of the following answers

Please choose **only one** of the following:

- Yes
- No
- Unknown

1. **Please choose the description(s) that best fit the position of the staff member(s) who had a positive test for COVID-19:** *

Please choose **all** that apply:

- Dentist
- Dental hygienist
- Dental assistant
- Receptionist
- Other:

1. **During this period did any of your co-workers, at the office you provided care, have any symptom which made you suspect that they have COVID-19?** *

Choose one of the following answers

Please choose **only one** of the following:

- Yes
- No
- Unknown

1. **Please choose the description(s) that best fit the position of the staff member(s) who had symptoms similar to COVID-19:** *

Check all that apply

Please choose **all** that apply:

- Dentist
- Dental hygienist
- Dental assistant
- Receptionist
- Other: _________________

# Section 6: COVID-19 Anxiety

1. **Please rate the extent to which each statement applies to you over the last two weeks**.*

|  | Not at all (0) | Rarely, less than a day or two (1) | Several days (2) | More than 7 days (3) | Nearly every day (4) |
| --- | --- | --- | --- | --- | --- |
| - I have avoided using public transport because of the fear of contracting coronavirus (COVID-19) |  |  |  |  |  |
| - I have checked myself for symptoms of coronavirus (COVID-19) |  |  |  |  |  |
| - I have avoided going out to public places (shops, parks) because of the fear of contracting coronavirus (COVID-19) |  |  |  |  |  |
| - I have been concerned about not having adhered strictly to social distancing guidelines for coronavirus (COVID-19) |  |  |  |  |  |
| - I have avoided touching things in public spaces because of the fear of contracting coronavirus (COVID-19). |  |  |  |  |  |
| - I have read about news relating to coronavirus (COVID-19) at the cost of engaging in work. |  |  |  |  |  |
| - I have checked my family members and loved one for the signs of coronavirus (COVID-19). |  |  |  |  |  |
| - I have been paying close attention to others displaying possible symptoms of coronavirus (COVID-19). |  |  |  |  |  |
| - I have imagined what could happen to my family members if they contracted coronavirus (COVID-19). |  |  |  |  |  |
| - I am afraid of getting COVID-19 from a patient or a co-worker |  |  |  |  |  |
| - I am anxious when providing treatment to patients with flu like symptoms |  |  |  |  |  |
| - I fear that the PPE I am using may not be sufficient to protect me against COVID-19 |  |  |  |  |  |

1. **Over the last 2 weeks, how often have you been bothered by the following problems ?***

|  | **Not at all (0)** | **Several days (1)** | **More than half the days (2)** | **Nearly every day (3)** |
| --- | --- | --- | --- | --- |
| - Feeling nervous, anxious or on edge |  |  |  |  |
| - Not being able to stop or control worrying |  |  |  |  |
| - Worrying too much about different things |  |  |  |  |
| - Trouble relaxing |  |  |  |  |
| - Being so restless that it is hard to sit still |  |  |  |  |
| - Becoming easily annoyed or irritable |  |  |  |  |
| - Feeling afraid as if something awful might happen |  |  |  |  |

# Section 7: Economic impact of COVID-19

Context:

The following set of questions are concerning the economic impact that the COVID-19 pandemic might have had on your practice and the anxiety you might have had due to any such economic impact during this period. Understanding that the situation may have been changing dynamically, we have divided the pandemic times into four periods as follows:

- Strict lockdown period (March to April 2020)
- Return to work period (May to July 2020)
- Chronic period (August to December 2020)
- Vaccination period (January 2021 to present)

Please refer to the above time periods in the pandemic while answering questions 3 to 6

1. **Which of the following options regarding type of dentist applies to you? ***

(Please choose all that apply)

- General dentist
- Specialist
- Practice-owner
- Associate dentist
- Employed with corporate dental practice
- Working in a hospital or dental school
- Other_______________________________

1. **Which practice-related factors have led to increased anxiety for you during the COVID-19 pandemic?***

(Please choose all that apply)

- Reduced revenue collection
- Ability to offer limited dental treatments
- Reduced number of patients
- Increased costs involved in practice
- Fear of losing your job
- Being redeployed to frontline healthcare services
- Laying off dental office staff
- Other_____________________________________

1. **Compared to before the pandemic, what was the change in your practice income during the following time periods of the COVID-19 pandemic?***

*‘Your practice income’* *refers to the income generated through services provided in your dental practice(s).*

|  | **Very**  **much decreased** | **Somewhat decreased** | **No change** | **Somewhat increased** | **Very much increased** |
| --- | --- | --- | --- | --- | --- |
| **Strict lockdown period**  *(Mar to Apr 2020)* |  |  |  |  |  |
| **Return to work period**  *(May to Jul 2020)* |  |  |  |  |  |
| **Chronic period**  (Aug to Dec 2020) |  |  |  |  |  |
| **Vaccination period**  (Jan 2021 to present) |  |  |  |  |  |

1. **Compared to before the pandemic, what was the change in your practice costs during the following time periods of the COVID-19 pandemic? ***

*Practice costs* *refers to the expenses involved in running the practice. These include the salaries paid to dental office staff.*

|  | **Very**  **much decreased** | **Somewhat decreased** | **No change** | **Somewhat increased** | **Very much increased** |
| --- | --- | --- | --- | --- | --- |
| **Strict lockdown period**  *(Mar to Apr 2020)* |  |  |  |  |  |
| **Return to work period**  *(May to Jul 2020)* |  |  |  |  |  |
| **Chronic period**  (Aug to Dec 2020) |  |  |  |  |  |
| **Vaccination period**  (Jan 2021 to present) |  |  |  |  |  |

1. **Compared to before the pandemic, what was the change in your practice net revenue generation during the following time periods of the COVID-19 pandemic? ***

*Net revenue is defined as “for you” only. It is income left over after practice expenses and business taxes and includes salary, commission, bonus and/or dividends and any payments made to a retirement plan on the dentist’s behalf.*

|  | **Very**  **much decreased** | **Somewhat decreased** | **No change** | **Somewhat increased** | **Very much increased** |
| --- | --- | --- | --- | --- | --- |
| **Strict lockdown period**  *(Mar to Apr 2020)* |  |  |  |  |  |
| **Return to work period**  *(May to Jul 2020)* |  |  |  |  |  |
| **Chronic period**  (Aug to Dec 2020) |  |  |  |  |  |
| **Vaccination period**  (Jan 2021 to present) |  |  |  |  |  |

1. **Compared to before the pandemic, what was the effect of the above economic aspects of your practice on your levels of anxiety during the following time periods of the COVID-19 pandemic? ***

|  | **Very**  **much decreased** | **Somewhat decreased** | **No change** | **Somewhat increased** | **Very much increased** |
| --- | --- | --- | --- | --- | --- |
| **Strict lockdown period**  *(Mar to Apr 2020)* |  |  |  |  |  |
| **Return to work period**  *(May to Jul 2020)* |  |  |  |  |  |
| **Chronic period**  (Aug to Dec 2020) |  |  |  |  |  |
| **Vaccination period**  (Jan 2021 to present) |  |  |  |  |  |

1. **Which factors have helped you to manage your anxiety during the COVID-19 pandemic? ***

(Please choose all that apply)

- Decided to retire earlier than previously planned
- Followed guidance from the provincial dental regulatory authority
- Submitted claims to dental insurance companies
- Received COVID-19 vaccination for yourself, family and dental office staff
- Enrolled in wellness initiative programs
- Financial support schemes from the government
- Other­­­­___________________________________________________________________

# Section 8: Overall impact of COVID-19

1. **What is your perspective on your regulatory body's COVID-19 guidelines?**
2. **Please provide any observations you have concerning the dental care provision during the COVID-19 pandemic:**

# Section 9: Participant satisfaction

*We would like to evaluate how you feel about this research study, for future research purposes.*

1. **Please select one answer that best represents how you feel about the online survey part of this research study***

|  | **Strongly agree (5)** | **Agree (4)** | **Neutral (3)** | **Disagree (2)** | **Strongly Disagree (1)** |
| --- | --- | --- | --- | --- | --- |
| 1. This research study met my expectations |  |  |  |  |  |
| 1. I was comfortable with the research procedure. |  |  |  |  |  |
| 1. I was comfortable working with the research team. |  |  |  |  |  |
| 1. The informed consent form was easy to understand |  |  |  |  |  |
| 1. Based on my experience with this research study, I would participate in a similar study in the future. |  |  |  |  |  |

1. **How disruptive was participating in this research study on your daily routine?***

- Completely disruptive
- Very disruptive
- Moderately disruptive
- Not very disruptive
- Not at all disruptive
