# Supplementary material for: Canadian dental hygienists’ experiences and perceptions of regulatory guidelines during the COVID-19 pandemic: a qualitative descriptive analysis
Source: BMC Health Serv Res. 2022 Dec 22;22:1570. doi: 10.1186/s12913-022-08925-z (PMC9773656; doi:10.1186/s12913-022-08925-z)
Supplement: Supplementary file 3 — Additional file 3. COREQ (Consolidated Criteria for Reporting Qualitative Research) 32-Item Checklist. [file 12913_2022_8925_MOESM3_ESM.doc]

**Supplemental 1.** COREQ (Consolidated Criteria for Reporting Qualitative Research) 32-Item Checklist

Canadian dental hygienists’ experiences and perceptions of regulatory guidelines during the COVID-19 pandemic: a qualitative descriptive analysis

| Domain | Item | Comments | Page Number or N/A |
| --- | --- | --- | --- |
| **Domain 1: Research team and flexibility** | | | |
| *Personal Characteristics* | | | |
| Interviewer/Facilitator | 1 | Web-based survey design, all authors are identified as conducting this research; LM and LR are described as facilitating intial review and thematic analysis of participant data. | Title page, 7-9 |
| Credentials | 2 | All authors hold credentials as oral health professionals and have research experience across qualitative and mixed-methods methodologies. | Title page, 7-8 |
| Occupation | 3 | Lead author (LM) and LR are practicing registered dental hygienists (RDH); MG, PA, CQ and SM, are dentists; all authors are experienced health researchers. | Title page, 7-8 |
| Gender | 4 | Two researchers identify as female; 4 identify as male. | Title page, 8 |
| Experience and Training | 5 | All authors are confirmed as having experience in qualitative research methodology. Lead author (LM) and co-author (LR) that lead primary data analysis and theme identification are confirmed as having experience in qualitative methods and thematic data analysis. | 7-8 |
| *Relationship with Participants* | | | |
| Relationship established | 6 | Anonymous web-based survey design; as such, the authors did not pre-establish relationships or affiliations with participants. Participant anonymity was maintained. Authors were blinded to participant identities throughout this study to mitigate risk of bias in the data that were collected. | 6, 8 |
| Participant knowledge of the interviewer | 7 | Participants were introduced to the authors, intentions of the research, rationale of the study and how their data would be used for the purpose of the research within the invitation to participate. | 8 |
| *Interviewer characteristics* | 8 | All authors described as having experience in qualitative research; facilitators of initial data analysis (LM and LR) described as trained in thematic analysis. | 7-8 |
| **Domain 2: Study Design** | | | |
| *Theoretical framework* | | | |
| Methodological orientation and Theory | 9 | Descriptive thematic analysis guided by combined techniques within qualitative tradition; commonly accepted QD study design. | 4, 5 |
| Participant selection | | | |
| Sampling | 10 | Participants were selected using prospective sampling methodology. | 4 |
| Method of approach | 11 | Registered dental hygienists were approached via email through their provincial regulatory body’s roster. | 6 |
| Sample Size | 12 | A total of 876 dental hygienists consented to participate in the longitudinal study; 400 responsed to Q1 (baseline); 247 responsed to Q2 (final follow-up). | 6 |
| Non-participation | 13 | Seventeen participants were excluded from participating due to circumstances outlined for exclusion (retirement; previous COVID-19 infection). | 6 |
| *Setting* | | | |
| Setting of data collection | 14 | Canada; participating provinces are described. Data were collected on a secure server housed behind firewall hosted by McGill University using LimeSurvey software . | 5,6 |
| Presence of non participants | 15 | N/A; authors cannot confirm presence of non-participants owing to anonymous web-based study design. | N/A |
| Description of sample | 16 | Sample characteristics are reported in Table 1 | 5 |
| *Data Collection* | | | |
| Interview guide | 17 | 76-question survey adapted from WHO Unit Study protocols for assessment of COVID-10 risk among health care workers; Open-ended question were developed by the research team, vetted and pilot-tested on a subset of participants. | 6-7 |
| Repeat interviews | 18 | No repeat interviews were conducted owing to study design. | N/A |
| Audio/visual recording | 19 | NA; no audio/visual recording devices used in study design or data collection. | N/A |
| Field notes | 20 | N/A; LM and LR wrote notes and reflected on themes identified in the textual data during thematic analysis. | N/A |
| Duration | 21 | Six surveys adminsistered bi-monthly for 12 months (Dec 2020-Jan 2022); Q1 responses collected at baseline (Dec 2020-Jan 2021) and Q2 responses collected at final follow up (Nov 2021-Jan 2022) | 6-7 |
| Data saturation | 22 | Data was reviewed by all research team members until no new themes were identified and saturation was determined. | 9 |
| Transcripts returned | 23 | N/A owing to study design | N/A |
| **Domain 3: Analysis and Findings** | | | |
| *Data Analysis* | | | |
| Number of data coders | 24 | LM and LR led initial review of data for themes; themes and sub-themes were reviewed and developed by all authors. | 8-9 |
| Description of the coding tree | 25 | Themes are described in the Results section | 9-18 |
| Derivation of themes | 26 | Themes were identified, reviewed and defined across initial theme identification and development (LM and LR) and research team meetings (review by all authors). | 8-9 |
| Software | 27 | Participant responses were extracted in their entirety from the LimeSurvey platform; identified themes were inserted into an electronic thematic spreadsheet. | 8-9 |
| Participant checking | 28 | Participants did not provide feedback on the identified themes owing to anonymity. | N/A |
| *Reporting* | | | |
| Quotations presented | 29 | Quotations that reflected or supported each identified theme were selected and included within the Results. | 9-18 |
| Data and findings consistent | 30 | There is consistency between data presented and findings. | 9-18 |
| Clarity of major themes | 31 | Major themes are clearly identified in Results and presented in Tables 2 and 3. | 11, 15 |
| Clarity of minor themes | 32 | Minor themes are clearly identified in Results and presented in Tables 2 and 3. | 11, 15 |

Developed from: Tong A, Sainsbury P, Craig J. Consolidated criteria for reporting qualitative research (COREQ): a 32-item checklist for interviews and focus groups. International Journal for Quality in Health Care. 2007. 19(6): 349 – 357.
